# Densimetry of diluted aqueous salt solutions and molecular dynamics simulations identify temperature-dependent differences between the hydration of anions and cations

**DOI:** 10.1038/s41598-025-14329-w

**Published:** 2025-08-08

**Authors:** Marta Onuk, Anna Stefaniuk, Iryna Doroshenko, Jarosław Poznański

**Affiliations:** 1 Institute of Biochemistry and Biophysics, Polish Academy of Sciences, Warszawa, Poland; 2 Faculty of Physics, Taras Shevchenko National University of Kyiv, Kyiv, Ukraine

**Keywords:** Density measurement, Partial molar volume, Temperature-dependence, Ion solvation, Kosmotropic and chaotropic effects, Molecular dynamics, Structure of solids and liquids, Thermodynamics

## Abstract

This study aims to analyze the temperature-dependent hydration of diluted ionic solutions. Three monovalent anions (Cl^-^, Br^-^, and I^-^), three monovalent cations (Li^+^, Na^+^, and K^+^), and one bivalent ion each (SO_4_^2-^ and Mg^2+^, respectively) were chosen as models. The partial molar volumes of all possible two-component salts (i.e., LiCl, NaCl, KCl, LiBr, NaBr, KBr. LiI, NaI, KI, MgCl_2_, MgBr_2_, MgI_2_, Li_2_SO_4_, Na_2_SO_4_, K_2_SO_4_, and MgSO_4_) were determined in water at low solute concentrations (10^− 3^ to 3·10^− 2^ mol/kg) in the 20 ÷ 40 °C temperature range. The density analysis was based on the first-order (linear) approximation of the density-molality relation corrected for the Debye-Hückel slope for volumes. No additional sophisticated corrections were applied. For all salts except the bivalent-bivalent MgSO_4_, the partial molar volume is positive and generally increases with temperature much more than bulk water. The temperature-dependent partial molar volumes of particular ions were determined globally, assuming the composition-dependent additive contribution to the partial molar volume of the salt. The qualitative differences between anions and cations were identified, reflecting their divergent electrostatic contributions to solute-solvent interactions. Similar nonlinear trends were observed in molecular dynamics simulations of the solvated separate ions at 10 ÷ 50 °C. The observed differences between anions and cations should be attributed to principal water properties, specifically the electron density distribution, which interferes with the packing of asymmetric water molecules around the ions of interest.

**Supplementary Information:**

The online version contains supplementary material available at 10.1038/s41598-025-14329-w.

## Introduction

Water is a liquid with unique properties determined by the dynamic network of hydrogen bonds formed between neighboring H_2_O molecules. Several theoretical models describe pure water’s properties, both implicit (e.g., SPT^1^, COSMO^2^, PCM^3^ and explicit ones, including the Mercedes-Benz^4^ and numerous multi-site forcefields commonly used in molecular dynamics simulations (MD) (including those developed from SPC^5^ or TIP3P^6^. However, the real challenge is to describe how a solute’s molecules interact with and affect the surrounding water molecules. In the case of non-polar compounds, this effect is known as hydrophobic solvation, a phenomenon for which Lum, Chanler, and Weeks proposed the most general theory in 2000^7^. On the other hand, the Debye-Hückel theory of electrolytes^8^, further extended with Pitzer’s Ion-Interaction Eq.^9^, is commonly used for ionic solutions.

Determining the thermodynamic parameters describing solvation is of great importance, especially when considering the medical applications of electrolytes^10–12^. Hydrogen bonds are also crucial in nanoscience, influencing the functionalization of surfaces with ordered molecular layers^13–15^. Recently, it has been reported that ions close to the water surface deviate significantly from the homogeneous distribution assumed in theoretical models of electrolytes^16^.

However, disregarding surface effects, it can be assumed that, once dissolved in water, electrolytes dissociate into cations and anions, distributing themselves regularly and uniformly in aqueous solution, altering the properties of the hydrogen bond network formed by proximal water molecules^17–19^. At low concentrations, electrolytes in water dissociate into hydrated ions, causing the dipolar water molecules to change the organization of the hydration shells around these ions, thus inducing structures that differ from bulk water in their packing and thermodynamic properties. Such a phenomenon is characteristic of water to such an extent that qualitative differences in the solvation of the HOD molecule in D_2_O and H_2_O have been identified^20^.

The effect of salts on hydrogen bonding has been the subject of active research since the late 19th century. From Hofmeister’s analysis of the impact of salts on physiological samples^21^ to Zena’s study of thermodynamic properties in 1956^22^, the structural effects of electrolyte interactions in solutions were studied. The detailed analysis of molar volume by Millero in 1971 and subsequent works by Lepori, Malatesta, Mao, Duan, Parmar, and others have contributed significantly to understanding the properties of electrolyte solutions^23–35^. More recently, attention has turned to explaining the effects of salts in terms of chaotropic and kosmotropic effects, describing how the different properties of ions affect the nature of hydrogen bonding between surrounding water molecules^36^. Collins highlighted the weaker interaction of chaotropes with water molecules compared to bonding water molecules to each other, while kosmotropes showed the opposite effect^37^. Chaotropism correlates with low charge density, making large monovalent ions chaotropic, while small or multivalent ions are predominantly kosmotropic^38,39^.

Volumetric studies of biomolecules in aqueous electrolyte solvents, elucidating biomolecule-electrolyte interactions, are becoming increasingly important. Understanding the thermodynamic properties of electrolyte solutions remains fundamental in various industrial applications, and concentration-dependent studies of apparent and partial molar volumes are proving valuable in elucidating structural interactions in solutions^40–44^.

**Fig. 1:**
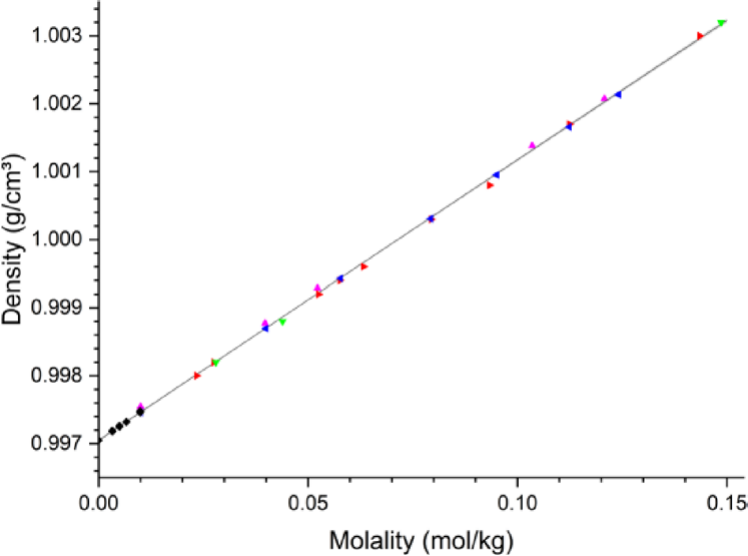
Density-molality relationship of NaCl in H_2_O at 25 °C. Colored triangles indicate the literature data^45–47^, and our study is by black diamonds. A fit to all the data presented according to Eq. 1 leads to the partial molar volume (V_2_^0^) being estimated to be 16.40 ± 0.12 cm³/mol.

Despite 150 years of research into the thermodynamic properties of salts in aqueous solutions, the general mechanism of this process is still not completely understood. However, limited information is available on the detailed structural behavior of ions in water at low concentrations (here < 0.03 mol/kg, Fig. 1). Consequently, assessing the relative importance of ion-water interactions resulting from electrolyte dissolution remains challenging.

As the partial molar volume of an electrolyte reflects the effects of ion-solvent, ion-ion, and solvent-mediated ion-ion interactions, it is interesting to study the partial molar volumes of anions and cations separately at such low concentrations that fulfill the Debye-Hückel approximation limits. Herein, we are using the approach we developed to analyze low-mass hydrophobic compounds with limited solubility^48,49^ in studies of diluted electrolyte solutions.

## Results

### Partial molar volumes (V_2_^0^) and the thermal expansion coefficients (α_2_^0^) of HCl and the sixteen salts in H_2_O

**Fig. 2:**
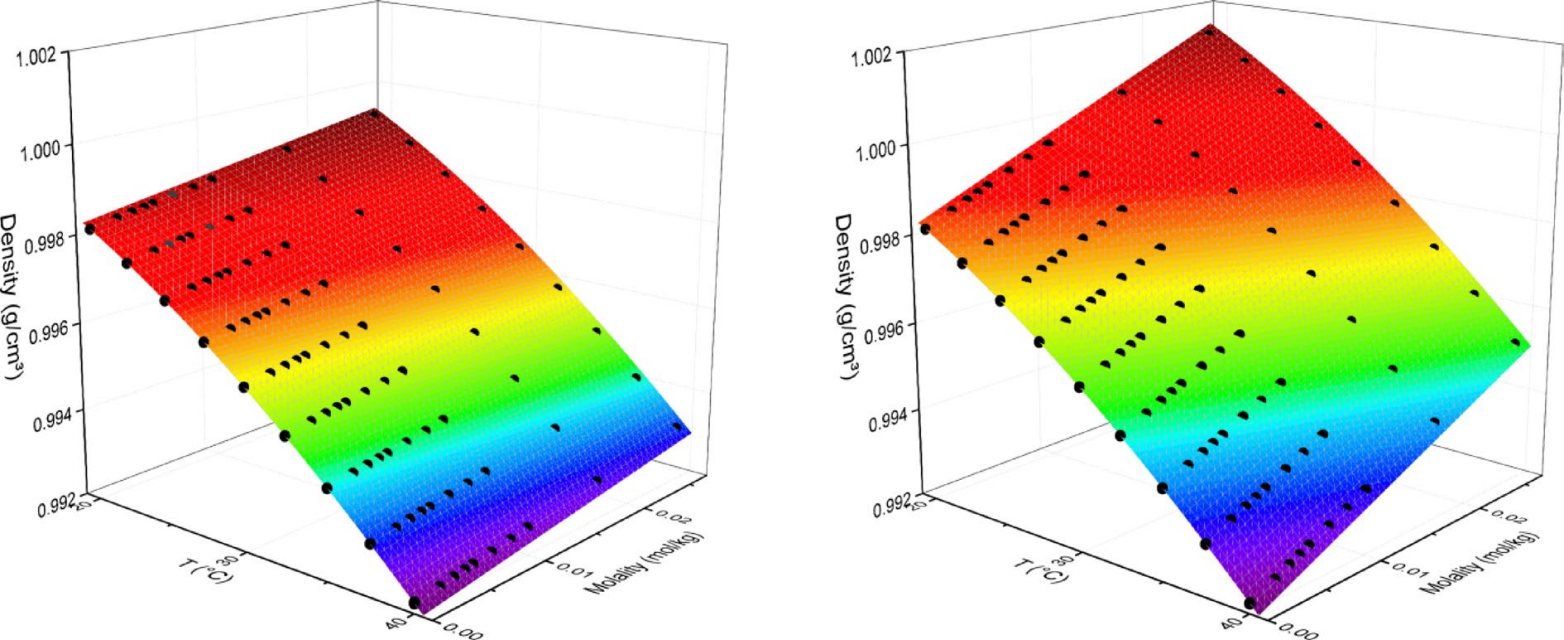
Global analysis of temperature- and molality-dependence of the density of aqueous solutions of NaCl and MgSO_4_. The data were collected at 20 ÷ 40 °C. The salt molal concentration varied between 10^− 3^ and 3·10^− 2^ mol/kg.

The experimental data on density for aqueous salt solutions at varying temperatures (20 ÷ 40 °C) are summarized in the Supplementary Data File. The density of electrolytic solutions in water is consistently higher than that of pure water at respective temperatures. In all cases, the linear density-molality relationship is observed, which validates the approximation of infinite dilution used in Eq. 1. After testing each salt, we assumed a quadratic approximation of the thermal expansion, as described by Eq. 2. The data collected at all temperatures were analyzed jointly for each salt using a global set of parameters characterizing a particular salt supplemented by a local parameter describing the deviation of the approximated bulk water density from the reference value^50^, Δd_o_(T), which value was estimated individually for each series of dilutions (see Fig. 2 for an example).

**Fig. 3:**
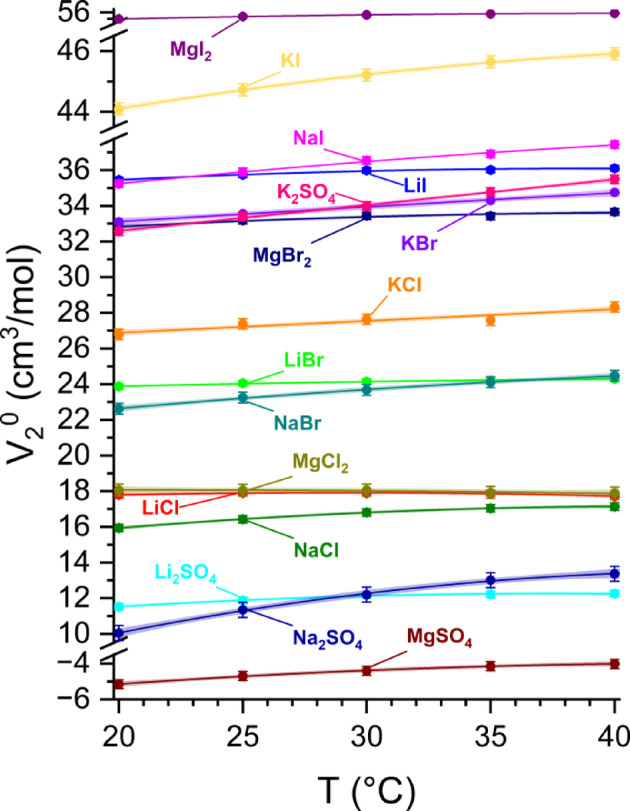
Temperature-dependence of the partial molar volume (V_2_^0^) of salts in water.

The temperature dependence of the partial molar volume of salts in water is shown in Fig. 3 and summarized in Table 1. It is worth mentioning that the asymptotic density of pure water, d_0_(T), agrees with the literature data; however, at a given temperature, individual estimates differ minutely (Δd_0_(T) < 2·10^− 5^ g/cm^3^).

All V_2_^0^ values, with the only exception of bivalent-bivalent MgSO_4_, are positive. All these values (see Table 1) are consistent with the literature data^22–32,51^. The monotonic increase of V_2_^0^ with temperature is observed for all salts except MgCl_2_. However, according to the quadratic approximation of thermal expansion, the maximum volume appears at 30–45 °C for the majority of the tested salts. It is worth noting that the thermal expansion coefficients estimated at 25 °C from the quadratic dependence V_2_^0^(T) exceed that of bulk water at the same temperature (4.6·10^− 3^ cm^3^/mol/K)^20^. The latter indicates that the organization of water molecules in the solvation shell is much more susceptible to temperature than in the bulk. We have previously reported a similar effect for HOD in D_2_O but not in H_2_O^20^, while such an effect was even stronger for hydrophobic dissociable solutes^49^.

**Table 1:** The partial molar volume (V_2_^0^), the thermal expansion coefficient (α_2_^0^), and the second-order correction (β_2_^0^, see Eq. 2) of the tested salts and HCl estimated at 25 °C from the density data, assuming either linear (upper row) or quadratic (bottom row) relation V_2_^0^(T).

Compound	V_2_^0^ [Ref.] cm^3^/mol	V_2_^0^ (25 °C) cm^3^/mol	α_2_^0^ (25 °C) 10^-3^cm^3^/mol/K	β_2_^0^ (25 °C) 10^-3^cm^3^/mol/K^2^	T_extr_ °C
LiCl	17.33^47^, 16.866^9^	17.85 (0.09) 17.89 (0.10)	-3 (10) 13 (20)	-1.6 (1.8)	31 (1)
NaCl	16.8^22^, 16.620^9^	16.37 (0.09) 16.43 (0.12)	61 (11) 87 (25)	-2.6 (2.2)	29 ( 2)
KCl	26.4^22^, 26.89^32^, 26.848^9^	27.19 (0.11) 27.22 (0.18)	61 (14) 66 (36)	0.0 (3.1)	42 ( 1)
MgCl_2_	15.6^22^, 14.083^9^	18.09 (0.13) 18.06 (0.20)	-10 (16) -4 (41)	-0.5 (3.5)	32 ( 1)
LiBr	24.08^47^ 22.758^9^	24.01 (0.08) 24.03 (0.09)	20 ( 9) 26 (18)	-0.6 (1.6)	38 (29)
NaBr	23.4^22^, 22.479^9^	23.16 (0.12) 23.21 (0.18)	89 (15) 106 (37)	-1.5 (3.2)	47 (11)
KBr	33.4^22^, 33.688^9^	33.53 (0.14) 33.54 (0.21)	77 (17) 83 (43)	-0.2 (3.7)	59 (10)
MgBr_2_	28.788^9^	33.18 (0.11) 33.15 (0.12)	40 (12) 55 (24)	-1.6 (2.1)	> 100
LiI	36.5^52^, 35.340^9^	35.70 (0.08) 35.75 (0.09)	32 ( 9) 50 (18)	-1.8 (1.6)	33 ( 2)
NaI	34.998^9^	35.86 (0.11) 35.90 (0.13)	108 (13) 123 (26)	-1.5 (2.2)	39 ( 4)
KI	45.48^53^, 44.95^54^, 45.5^47^, 45.151^9^	44.66 (0.11) 44.71 (0.12)	91 (12) 113 (24)	-2.2 (2.1)	65 (19)
MgI_2_	50.174^9^	54.62 (0.14) 54.72 (0.16)	89 (16) 129 (32)	-4.1 (2.7)	51 ( 3)
Li_2_SO_4_	12.68^55^,12.580^9^	11.83 (0.10) 11.89 (0.11)	36 (11) 60 (22)	-2.4 (1.9)	41 ( 3)
Na_2_SO_4_	10.9^22^, 11.54^56^, 11.776^9^	11.20 (0.17) 11.30 (0.25)	171 (21) 221 (52)	-5.5 (4.5)	37 ( 2)
K_2_SO_4_	30.1^22^, 32^32^, 32.050^9^	33.37 (0.10) 33.32 (0.14)	142 (12) 145 (28)	-0.1 (2.5)	45 ( 3)
MgSO_4_	-3.9^22^, -3.4^56^	-4.74 (0.10) -4.71 (0.11)	58 (12) 73 (25)	-1.5 (2.2)	> 100
HCl	18.07^57^, 17.824^9^	17.70 (0.05) 17.69 (0.07)	6 ( 6) 21 (13)	-1.7 (1.2)	45 ( 3)

### Partial molar volume (V_2_^0^) of the eight studied ions

To meet with the analysis of Marcus^58^, Krumgalz et al.^9^, or Millero^31^, we have estimated partial molar volumes of the particular ions, assuming their additive contribution to the experimental partial molar volumes of the resulting salt. The set of partial molar volumes of particular ions was estimated for all studied temperatures using molality-density data determined for sixteen salts (Table 2). Such analysis was supplemented with data for HCl solutions and additional constraints for hydrated proton applied to overcome the problem of an undefined system of equations characterized by a zero-determinant matrix. However, significant discrepancies exist between the estimates for the proton partial molar volume in H_2_O at 25 °C, V_2_^0^(H^+^), reaching a few cm^3^/mol. The most reliable value is that of Borsarelli and Braslavsky^59^, V_2_^0^(H^+^) = (-5.5 ± 0.8) cm^3^/mol, in agreement with data obtained from conventional methods^22,60^, and we have applied the equation proposed for the volume of aqueous hydrogen by Marcus^61^:

$$V_{2}^{0} \left( T \right) = ~ - 5.1 - 0.008\times\:T - 1.7\cdot10^{{ - 4}}\times\:T^{2}$$

where V_2_^0^ is expressed in cm^3^/mol and T is in °C.

**Table 2:** The self-consistent set of partial molar volumes (V_2_^0^), based on density data measured for HCl and sixteen salts, estimated for eight ions at 20 ÷ 40 °C using the quadratic approximation of the temperature dependence. The standard errors are denoted in parentheses.

T, °C	V_2_^0^, cm^3^/mol								
	H^+^^61^	Li^+^	Na^+^	K^+^	Mg^2+^	Cl^-^	Br^-^	I^-^	SO_4_^2-^
20	-5.33	-5.44 (0.22)	-6.20 (0.24)	4.32 (0.24)	-27.07 (0.43)	22.67 (0.18)	29.68 (0.23)	40.59 (0.23)	22.63 (0.45)
25	-5.41	-5.56 (0.21)	-5.91 (0.22)	4.48 (0.22)	-27.26 (0.40)	22.85 (0.16)	29.96 (0.21)	41.06 (0.21)	23.24 (0.41)
30	-5.49	-5.72 (0.20)	-5.74 (0.22)	4.52 (0.22)	-27.59 (0.39)	23.03 (0.16)	30.25 (0.21)	41.48 (0.21)	23.81 (0.41)
35	-5.59	-5.90 (0.21)	-5.57 (0.22)	4.65 (0.22)	-27.86 (0.41)	23.09 (0.17)	30.41 (0.22)	41.75 (0.22)	24.28 (0.42)
40	-5.69	-5.91 (0.25)	-5.32 (0.26)	5.01 (0.26)	-27.81 (0.47)	23.07 (0.19)	30.51 (0.25)	41.83 (0.25)	24.30 (0.49)
data from^58^		-6.29	-6.62	3.61	-31.99	23.24	30.12	41.63	24.80
∂V/∂T ^(a)^ 10^− 3^ cm^3^/mol/K	-16.5	-31.8 (5.5)	44.7 (5.9)	17.1 (5.9)	-57.6 (10.6)	35.8 (4.3)	57.2 (5.6)	89.9 (5.6)	124.4 (11.1)
∂^2^V/∂T^2^ 10^− 3^ cm^3^/mol/K^2^	-0.17	0.56 (0.49)	-0.30 (0.51)	1.31 (0.51)	1.49 (0.93)	-1.50 (0.38)	-1.47 (0.50)	-2.62 (0.49)	-3.61 (0.97)
T_extr_, °C	-24	53 (20)	> 100	18 (5)	44 (9)	37 (2)	44 (5)	42 (2)	42 (3)

(a) estimated at 25 °C.

**Fig. 4:**
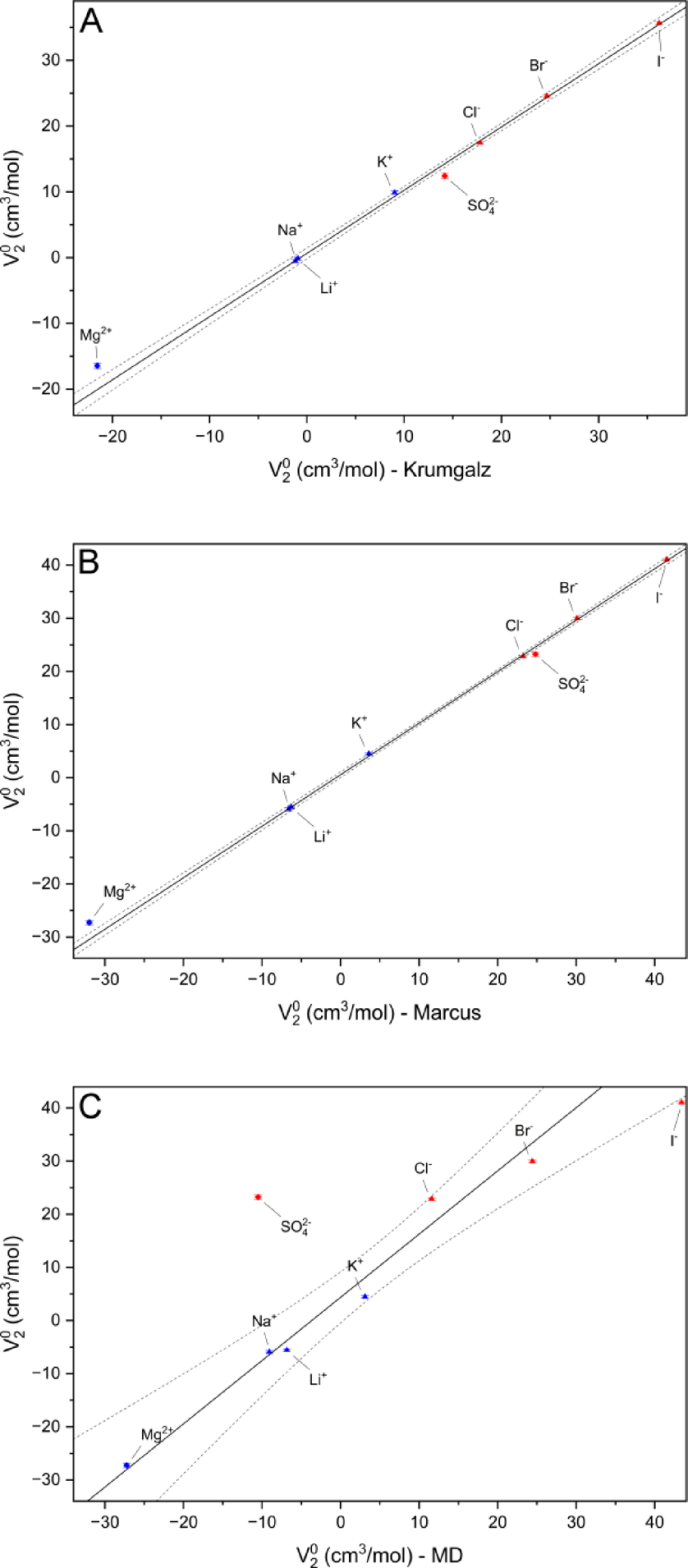
Correlation between the estimated self-consisted set of partial molar volumes (V_2_^0^) determined at 25 °C with the literature data: (**A**) published by Krungalz^9^, (**B**) published by Marcus^58^, and (**C**) with the MD-derived partial molar volumes.

Comparing data with Marcus, we have assumed that H^+^ contributes by -5.41 cm^3^/mol at 25 °C. All the obtained values are close to those estimated by Marcus^58^ and consistent with the values published by Krumgalz et al.^9^ or Millero^31^, with the only exception of Mg^2+^, the partial molar volume of which remains overestimated by 5.1, and 4.7 cm^3^/mol relative to the literature data, respectively, when the V_2_^0^ of all other ions differs by ~ 0.5 cm^3^/mol, see Fig. 4A, B for details. The standard error for the partial molar volumes estimated for monovalent ions is app. 0.2 cm^3^/mol and ~ 0.4 cm^3^/mol for the bivalent ones. All these values are within the uncertainty of the V_2_^0^ values determined separately for each salt, so it could be expected that the proposed approach yields a self-consistent set of volumetric parameters. The observed discrepancies with the values proposed by Marcus must result directly from neglecting in our analysis all other than Debye–Hückel corrections experimentally–derived parameterization, the contribution of which in Pitzer’s approach increases for bivalent ions. Such a correction remained negligible for all studied low-concentration solutions.

Among the first-order thermal expansion coefficients (i.e., ∂V_2_^0^/∂T), special attention is worth Li^+^ and Mg^2+^, which display negative values at 25 °C, indicating a decrease in the volume of these two ions with increasing temperature. Such an effect may correlate with the kosmotropic properties of these cations, which are characterized by high charge density. However, the most interesting finding concerns the second-order coefficients, a sign of which depends on the nature of the anion – it is negative for all anions studied and positive for all cations. Since such an effect has not been reported, we performed a more comprehensive analysis, estimating in silico a separate ion volume over a wider temperature range of 10 ÷ 50 °C.

### In silico analysis of ion solvation

According to the naïve hydration model, water molecules solvating a cation turn towards it with oxygen atoms, unlike those solvating an anion, for which hydrogen atoms are directed toward the ion. However, in the latter case, the shape of the water molecule makes the packing within the first solvation spheres with hydrogen atoms pointing toward the central anion less efficient. So, due to such a simple steric effect, anions have a molecular molar volume larger than cations of comparable ionic radius. In both cases, an increase in temperature will cause a partial randomization of the orientation of the solvating water molecules. Then, as the temperature increases, the packing of the cations’ solvation shell becomes less optimal, while the opposite effect can be expected for anions. A simple packing effect may explain opposite temperature dependences deduced for cations and anions from density measurements of salt aqueous solutions. Such an effect could, however, be studied in silico. We analyzed ion solvation using the 3-site TIP3P water parameterization^6^, a reliable water model commonly used in the in silico analyses of biological systems.

#### 2-D density maps of the solvation shell

**Fig. 5:**
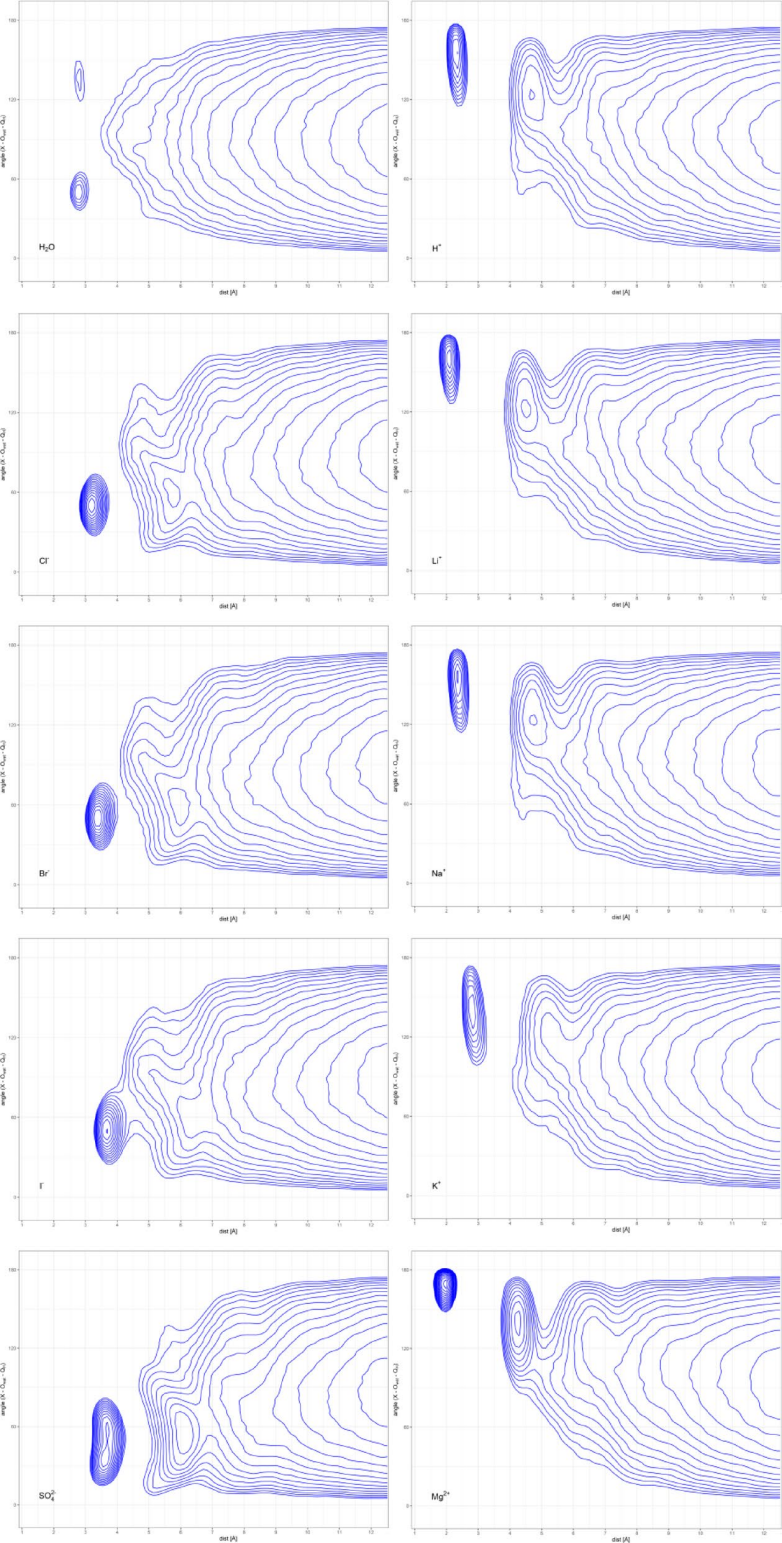
Uncorrected density maps derived from 10^4^ snapshots of 10 ns MD simulations of the system of an object of interest solvated by app 500 water molecules. The blue levels display the uncorrected water density in the space defined by the distance between the center of mass of the water molecule (Q), and the solvated ion (dist) and the ion-O_wat_-Q angle.

For each water molecule, the orientation relative to the solvated ion was defined by the distance between that ion (sulfur for SO_4_^2-^ and oxygen for H_2_O) and the water oxygen, ***d***, and the angle formed by a solute, water oxygen, and water center of mass, ***θ***. Together, they define locally the spherical coordinate systems centered at each water molecule. Compared to the standard radial density analysis, such an approach provides additional information about the effect of the solvated molecule on the distance-dependent orientational preferences of water molecules. We have analyzed ten systems: eight consisting of a separated ion of interest, and two reference ones, namely hydrogen cation and bulk water (Fig. 5). The obtained d-θ maps, which represent the distribution of water molecules around the ion, enable a qualitative analysis of the hydration phenomena. Thus, in the case of bulk water, two preferred orientations of water molecules in the first solvation sphere can be observed - corresponding to species that are donors (θ > 120°) or acceptors (θ < 60°) of a hydrogen bond. Interestingly, the orientation of a water molecule as the donor of a hydrogen bond is much better defined than when it is the acceptor (Supp. Figure 1). However, this is a direct consequence of the potential in the water model used. It is also still possible to isolate a trace of the second and third solvation spheres, in which water is already oriented almost randomly being distributed closely to the expected uncorrected density in the asymptotic form of d^2^·sin(θ). In the case of the hydrogen cation, the first two hydration shells are visible, for which the single maxima at large θ values indicate water oxygen oriented toward the hydrated cation; however, in the second solvation shell, a minute population with randomly oriented water molecules is also visible. The preferred orientation also dominates in the third shell; however, water molecules in planar orientation (θ ≈ 90°) or even those with one hydrogen atom pointing toward a cation (θ ≈ 60°) could also be observed. The analogous maps were obtained for the three monovalent cations tested, Li^+^, Na^+^, and K^+^, which mainly differ in the size of particular solvation shells, the locations of which follow the increase in the ionic radii in the order Li^+^ < Na^+^ < K^+^. As expected, different preferences are observed for the solvation of monovalent ions, Cl^-^, Br^-^, and I^-^, for which the first solvation shell is predominated by water molecules with the hydrogen atom pointing toward a solvated anion (θ ~ 60°). Notably, the second solvation shell is irregularly distributed in the map, and bending the d(θ) relation compensates for a suboptimal packing of water molecules in the first solvation shell. Such an effect has not been reported yet, as it remains invisible when the two marginal distributions - radial density and angular distribution (asymptotically converging to a uniform one) are analyzed separately. Finally, the third solvation shell can also be identified. The maps obtained for bivalent ions resemble those for their monovalent analogs. However, their kosmotropic character makes the first three solvation shells much better resolved, with the fourth one additionally evidenced. It is worth noting that for SO_4_^2-^ two slightly differing water orientations in the first solvation shell could be easily distinguished and that heterogeneity propagates to the second solvation shell.

#### Partial molar volume of ions and its temperature dependence

The above in silico analyses qualitatively reproduced the expected properties of bulk water and aqueous solvent solvating mono- and bivalent ions. Therefore, we focused on the force field’s accuracy in reproducing the density-derived parameters, specifically the partial molar volumes of ions and their temperature dependence. For each ion, we analyzed the cumulative radial distribution of water molecules averaged over 10^4^ MD snapshots at a temperature range of 10 ÷ 50 °C. We assumed that the number of water molecules contained by a sphere, N, is asymptotically proportional to the volume of that sphere, V. When the sphere is centered at the ion of interest, the distribution of proximal water molecules (those forming solvation shells) is perturbed. So, when a few proximal water molecules are excluded from the analysis (we disregarded 35 molecules closest to the ion), the slope of a linear approximation V(N) represents the V_2_^0^ of the water molecule in bulk, and the intercept states the estimation of the partial molar volume of the ion of interest. We have fitted all the data globally, assuming the common value of the slope, which represents the universal V_2_^0^ of water in bulk. At 20 °C, the slope of 30.023(1) Å^3^ corresponds to a bulk water density of 0.9968 g/cm^3^, close to the reference value of 0.9982 g/cm^3^. The V_2_^0^ values estimated for ions are close to those deduced from density measurements; however, the MD-derived V_2_^0^ for SO_4_^2-^ is substantially biased, which may indicate that oxygen VdW radius in AMBER14 parameterization should be corrected for SO_4_^2-^ (Fig. 4C).

## Discussion

**Fig. 6:**
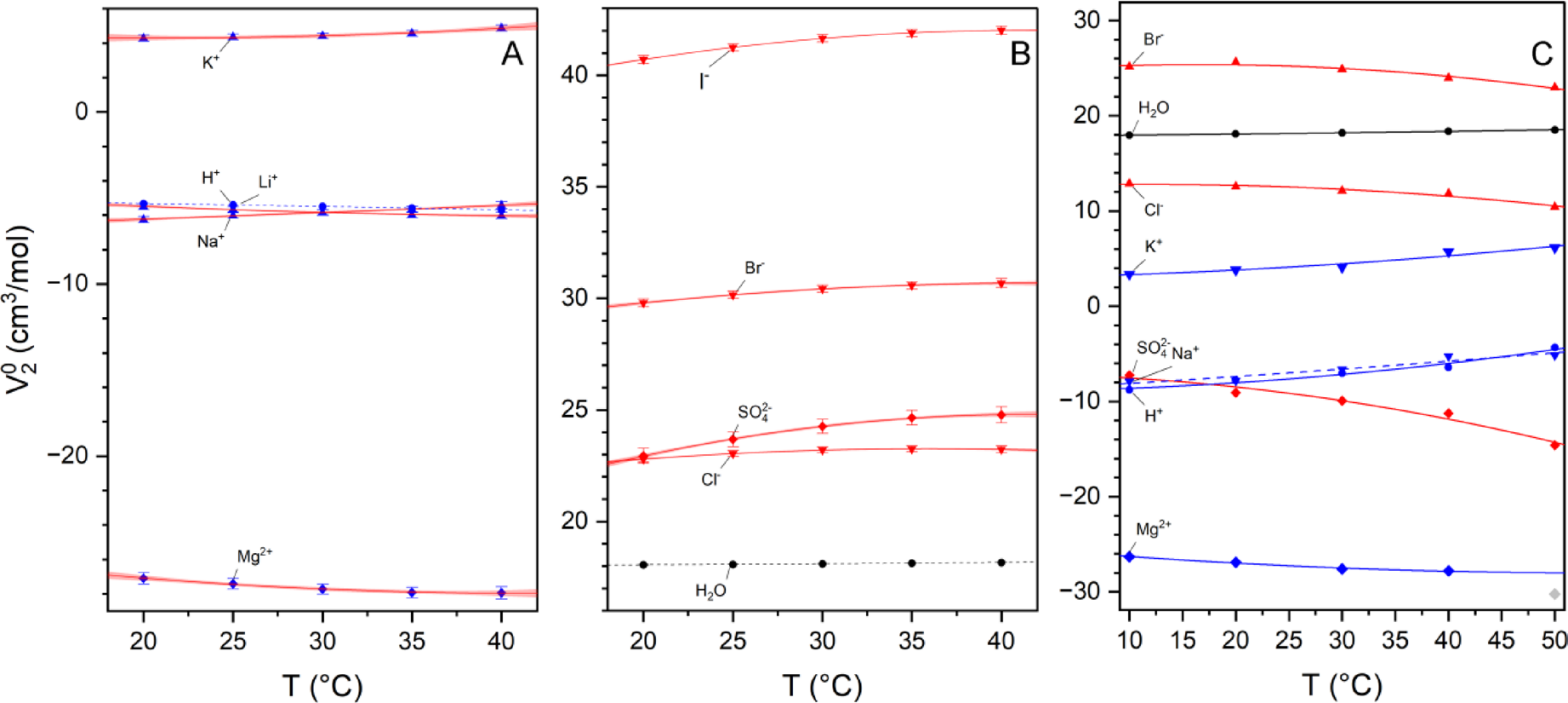
Temperature dependence of the estimated partial molar volumes (V_2_^0^) of the analyzed ions. The presented values were estimated based on a global analysis of over 800 density measurements. The data for H^+^ were taken from the literature^61^. Cations are denoted in blue and anions in red; monovalent and bivalent ions are represented by triangles and diamonds, respectively; gray regions mark 95% confidence bands for the fitted curves; the data for water (black circles) is shown for reference. Two left panels display experimental values for cations (**A**) and anions (**B**), respectively. The right panel (**C**) presents the MD-derived data.

The comparison of the global values with the volumes deduced from ionic radii reported by Marcus^62^ shows that for all monovalent anions, the experimental partial molar volumes (both presented here and those reported by Marcus) could be predicted from their ionic atomic radii. The partial molar volume of all ions is smaller than the value deduced from ionic radii taken from crystalographic data, while the opposite effect is observed for hydrophobic neutral species^48,63–65^. Strong negative values observed for magnesium (and other bivalent cations) indicate a sizeable kosmotropic effect. A weaker kosmotropic effect is observed for proton, lithium, and sodium. In the case of monovalent ions, the contribution of the hydration effect, including electrostatic ion-water interactions, remains uniform and possibly does not critically depend on the applied model (here, Pitzer-type with a parametrization adapted from^66^ – see Eq. 1). For bivalent ions, that correction becomes larger as ω is increasing. So, the Debye-Hückel slope’s contribution depends much more on the particular parameterization (i.e., Av values). We have assessed the effect of the Debye-Hückel correction, which, within the used narrow range of concentrations, contributes effectively by 0.26, 1.25, and 1.90 cm^3^/mol to the V_2_^0^ estimates for monovalent-monovalent, monovalent-bivalent, and bivalent-bivalent salts, respectively. Another possible factor that affects the estimated values is the concentration range at which Eq. 1, or its extension with higher-order corrections for apparent volume vs. molality relationship (V_φ_(m)), is applied. In principle, the higher the concentration range, the more significant the contribution of higher-order corrections will be. Since all those parameters are correlated upon fitting, the higher concentration range makes the V_2_^0^ estimate more precise (in terms of the standard error) but possibly more biased due to the propagation of additional factors used in data analysis. Our approach of the global analysis of volumetric data determined within a narrow concentration range of 10^− 3^ ÷ 3·10^− 2^ mol/kg optimizes the analysis of density data with Debye-Hückel correction for ion-solvent interactions without any correction for solute-solute interactions. Such an approach implies that any additional parameter were to be fitted. Finally, the proposed approach enabled the reliable quadratic approximation of the temperature dependence of the partial molar volumes of tested eight ions. The temperature dependence of the partial molar volume of studied anions and cations is shown in Fig. 6, and the data are summarized in Table 2.

The proposed analysis shows how the partial molar volume varies with the temperature. The most distinguishing factor is the trend observed in a quadratic approximation of the V_2_^0^(T) relationship. Thus, the curves in Fig. 6 are convex for all metal cations, while the opposite concave shape is observed for anions. Such discrepancy must reflect differences in the entropy-enthalpy balance of ion solvation, which is additionally affected by a solvation sphere’s heat capacity change. Solvation of both types was reported to differ in their enthalpic^67^ and heat capacity (ΔCp)^68^ contributions, which must reflect the temperature dependence of the solute-solvent electrostatic interactions. It should be emphasized that no such effect has been reported to date. Interestingly, pure water and apparent H^+^ variations are much tinier, identifying that proton solvation only minutely affects the solvation shell’s properties, which resemble those of bulk water.

We have proposed a protocol that allows the same volumetric parameters to be extracted from MD trajectories. These data also reveal the qualitative differences in the temperature dependence of the solvation of anions and cations, respectively (Fig. 6). The observed trends (curvatures) generally agree with the relations deduced from experimental density data.

Finally, we demonstrated that the parameterization of the sulfate ion in the AMBER14 forcefield must be improved. Moreover, the proposed approach can be applied to any water model, so it serves as an additional experimental method for verifying advanced water models.

## Materials and methods

### Sample preparation

The anhydrous magnesium sulfate (MgSO_4_; M = 120.36 g/mol; CAS-No: 7487-88-9), sodium sulfate (Na_2_SO_4_; M = 142.37 g/mol; CAS-No: 7757-82-6), potassium sulfate (K_2_SO_4_; M = 174.259 g/mol; CAS-No: 7778-80-5), magnesium chloride (MgCl_2_; M = 95.211 g/mol; CAS-No: 7786-30-3), sodium chloride (NaCl; M = 58.44 g/mol; CAS-No: 7647-14-5), potassium chloride (KCl; M = 74.555 g/mol; CAS-No: 7447-40-7), magnesium bromide (MgBr_2_; M = 184.113 g/mol; CAS-No: 7789-48-2), sodium bromide (NaBr; M = 102.894 g/mol; No: 509 − 56) and potassium bromide (KBr; M = 119.002 g/mol; BN-65/6191-40) were purchased from either Sigma-Aldrich (www.sigmaaldrich.com) or POCH (www.poch.com.pl) with the highest available purity (p.a.). Before preparation, each salt sample was kept for 1 h at 300 °C to remove the remaining residual water. The stock solutions were then prepared by mass using water demineralized and filtered with an ELIX system (Millipore).

The residual concentration of dissolved air was assessed by comparing the density-temperature relationship of the Millipore water sample with the data obtained for the Liquid Density Standard Ultra Pure Water sample provided by Anton Paar. In the temperature range of 20 ÷ 35 °C, the observed difference was 36 ± 2 · 10^− 6^ g/cm^3^. The latter value leads to an estimated N_2_ concentration of 0.13 mM, assuming a V_2_^0^ of 33.1 cm³/mol^69^ and a molecular mass of 28.014 g/mol, or 0.04 mM for O_2_, respectively. The density of the Millipore water sample degassed with argon was found between that of the Millipore and ultra-pure water samples (16 ± 2 · 10^− 6^ g/cm^3^). Bearing in mind that the tested solute concentrations were 10 to 100 times higher than the estimated residual air concentration, we decided to perform all the measurements with non-degassed Millipore water.

In the case of hydrochloric acid (HCl; M = 36.45 g/mol; CAS-No: 7647-01-0), the reference 100 mM aqueous solution was used to prevent handling concentrated acid solutions; however, we tested this solute in the same concentration range as salts.

### Density measurements

For each preparation, the densities for a series of the dilutions of the stock solution were determined with a high-precision density meter Anton Paar DMA 5000 M (declared precision 7·10^− 6^ g/cm^3^) in the temperature range of 20 ÷ 40 °C with 5 °C increment (declared precision better than 0.02 °C). The instrument was routinely calibrated for the used Millipore water sample at 20 °C against the literature density of the pure solvent in normal conditions^50^. The water density in the tested temperature range deviated from the reference data less than the declared precision (7·10^− 6^ g/cm^3^), which is consistent with the declared temperature repeatability of 0.001 °C. A molal concentration of a solute varied between 10^− 3^ and 3·10^− 2^ mol kg^− 1^.

The partial molar volumes ($\:{V}_{2}^{0}$) of the analyzed systems (MgSO_4_, Na_2_SO_4_ K_2_SO_4_, MgCl_2_, NaCl, KCl, MgBr_2_, NaBr, KBr, and HCl in H_2_O) were estimated from at least three independent dilution series based on the first-order approximation of the density–molality relationship^48,64^ corrected for the Debye-Hückel theoretical slope for volumes according to Pitzer-type equation^70^:

1 $$\:{V}_{2}^{0}+{A}_{v}\times\:{\omega\:}\times\:\text{l}\text{n}(1+b\times\:\sqrt{\omega\:\times\:m})/b=\frac{M}{{d}_{0}}-\frac{{10}^{3}}{{d\:\times\:d}_{0}}\times\:\frac{{d-d}_{0}}{m}$$

where *M* is the molar mass of solute (g/mol) calculated using the recent IUPAC recommendations^71^, m is the molal concentration of a solute (mol/kg), and d and d_o_ are the experimental density of the solution, and the pure solvent (g/cm^3^), respectively. $$\:{A}_{v}$$ is the temperature-dependent Debye-Hückel slope for volumes (the values were taken from^66^; however, we have checked that the data taken from^72^ negligibly affected the resulting $$\:{V}_{2}^{0}$$ estimates), ω is the valence factor ($$\omega = \raise.5ex\hbox{$\scriptstyle 1$}\kern-.1em/ \kern-.15em\lower.25ex\hbox{$\scriptstyle 2$} ~\sum n_{i} z_{i}^{2}$$), and b was set to be 1.2·10^− 3^ (g/mol)^½^^70^.

The numerical model based on Eq. 1 was implemented in Origin 10.0 (www.originlab.com). For each salt, the model parameters were fitted globally to all dilution series at each temperature. However, following our previous analyses of hydrophobic compounds^49^, we found that according to both Akaike’s Information Criterion and F-test, the model that assumed quadratic approximation of thermal expansivity of the solute, ($\:{{\alpha\:}_{2}^{0}=\partial\:V}_{2}^{0}/\partial\:T$, $\:{\beta\:}_{2}^{0}={{\partial\:}^{2}V}_{2}^{0}/\partial\:{T}^{2}$) was scored better than that with linear approximation ($\:{\beta\:}_{2}^{0}=0$). So, each salt is characterized by its partial molar volume ($\:{V}_{2}^{0}$) at a reference temperature of 25 °C and the temperature-independent apparent thermal expansivity coefficients ($\:{\alpha\:}_{2}^{0},\:{\beta\:}_{2}^{0}$). Such an approximation was proven valid, at least within the 20 ÷ 40 °C temperature range, and for all individual salts.

2 $$\:{V}_{2}^{0}\left(T\right)={V}_{2}^{0}\left({T}_{ref}\right)+{\alpha\:}_{2}^{0}\times\:\left({T-T}_{ref}\right)+{\beta\:}_{2}^{0}\times\:{\left({T-T}_{ref}\right)}^{2}$$

### Partial molar volumes (V_2_^0^) of ions

We used so diluted solutions that solute-solute interactions remained negligible, as experimentally validated by a linear molality-density relationship. Applying the first-order approximation, we assumed additive contributions of all ions to which dissociate a salt. We additionally assumed that the partial molar volume of a particular ion remains unaffected by salt composition. Upon such assumptions, the partial molar volume of any dissociable solute (X_n_Y_m_) is determined by the partial molar volumes of its ionic components (X, Y) as follows:

3 $$\:{V}_{2}^{0}\left({X}_{n}{Y}_{m}\right)=n\times\:{V}_{2}^{0}\left(X\right)+m\times\:{V}_{2}^{0}\left(Y\right)$$

where $\:{V}_{2}^{0}$ can be estimated by combining Eqs. 1 and 2.

Volumetric data was determined for sixteen salts composed of four different cations (Li^+^, Na^+^, K^+^, and Mg^2+^) and four anions (Cl^−^, Br^−^, I^−^, and SO_4_^2−^). Such an approach yields a system of sixteen linear equations with eight unknown variables, which should formally support valuable estimation of the partial molar volumes for all ions of interest. Unfortunately, the determinant of that system is zero, so we required an additional constraint, which was the density data for HCl, supplemented by volumetric data for hydrated proton taken from the literature^61^. We also applied second-order approximation for $\:{V}_{2}^{0}\left(T\right)$, i.e., assumed that $\:{\beta\:}_{2}^{0}\ne\:0$.

### Ion solvation analyzed with molecular dynamics simulations

All simulations were conducted with the Yasara Structure package^73^ using the TIP3P water model from the AMBER14 forcefield^74^, which was automatically modified for monovalent^75^ and bivalent^76^ ions. The molecular dynamics simulations were conducted with an isothermal-isobaric ensemble (NTP) in a cubic box of 25 Å edges with periodic boundary conditions and an 8 Å cutoff for electrostatic interactions. For each ion, the single ion was placed in the center of the cube, further filled with water molecules (507 ± 2, the exact number depended on the ion), and subjected to 100 ns MD; ten thousand snapshots (every 10 ps) were taken and analyzed with Yasara Structure as follows: for every snapshot, water molecules within 12.5 Å sphere of the ion were identified (in the case of SO_4_^2−^, the distance was measured relative to the sulfur atom). For each of them, the distance ion-O_wat_ (***d***) and the angle ion-O_wat_-Q_wat_ (***θ***) were recorded to describe the orientation of a water molecule relative to the ion (Q_wat_ is the center of mass of water hydrogens). The 2D distributions of water molecules solvating ion were visualized with R package^77^ using ggplot2 library^78^ to supplement ***θ*** vs. ***d*** scatterplots with 2D-density maps.

Partial molar volumes of ions were estimated as the intercept of the linear relation *V(N)*, where V was estimated from the MD trajectory as the mean volume limited by a sphere bordering *N* water molecules closest to the analyzed ion. The slope, corresponding to the volume of a water molecule in a bulk solvent, was fitted globally to all trajectories recorded at a given temperature. The latter assumption implies that bulk water density does not depend on the ion properties.

## Conclusion

With global analysis of sixteen diluted electrolyte solutions (m ≤ 2·10^− 3^ mol/kg), we determined a self-consistent set of partial molar volumes for Li^+^, Na^+^, K^+^, Mg^2+^, Cl^−^, Br^−^, I^−^, and SO_4_^2−^ at 20 ÷ 40 °C. The convex temperature dependences for cations significantly differ from the concave ones observed for anions (*p* < 10^− 3^). No such difference has already been reported for volumes of simple ions; however, other ion solvation parameters (enthalpy and heat capacity) differ for both ion types^67,68^.

## Supplementary Information

Below is the link to the electronic supplementary material.

Supplementary Material 1

## Data Availability

Data is provided within the manuscript or supplementary information files. The density datasets determined and analysed during the study are available from the corresponding author on reasonable request. The unprocessed distributions extracted from MD trajectories are available from the corresponding author on reasonable request.
