# Supplementary material for: Densimetry of diluted aqueous salt solutions and molecular dynamics simulations identify temperature-dependent differences between the hydration of anions and cations
Source: Sci Rep. 2025 Aug 8;15:29005. doi: 10.1038/s41598-025-14329-w (PMC12334640; doi:10.1038/s41598-025-14329-w)
Supplement: Supplementary file 1 — Supplementary Material 1 [file 41598_2025_14329_MOESM1_ESM.pdf]

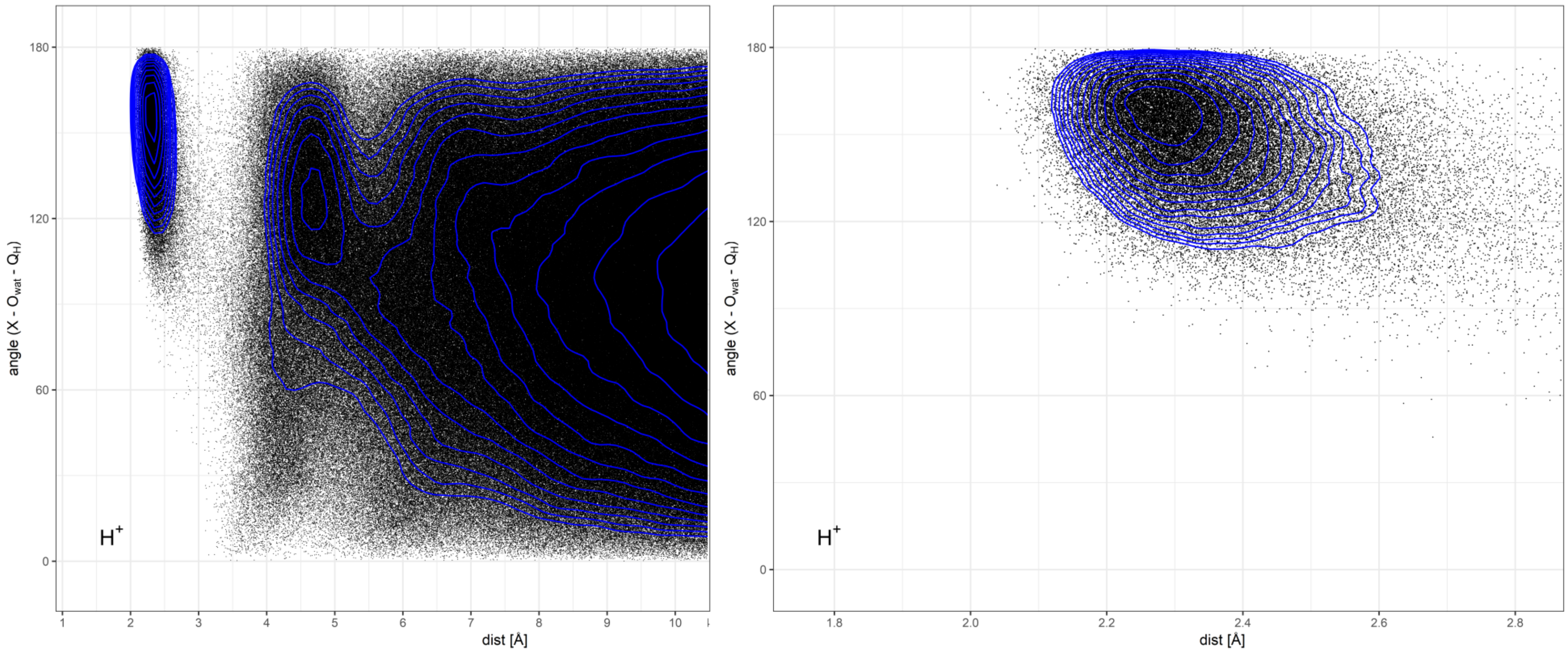

Supp. Figure 1A Uncorrected density maps derived from 10^4 snapshots of 10ns MD simulations of the system, consisting of a H^+ cation solvated by app 500 water molecules. Each dot represents the relative orientation of a single water molecule, defined by the distance between the center of mass of the water molecule (Q) to the solvated ion (dist) and the ion-O_{wat}-Q angle. The blue levels display the uncorrected water density in that space. The right panel zooms on the region corresponding to the first solvation shell.

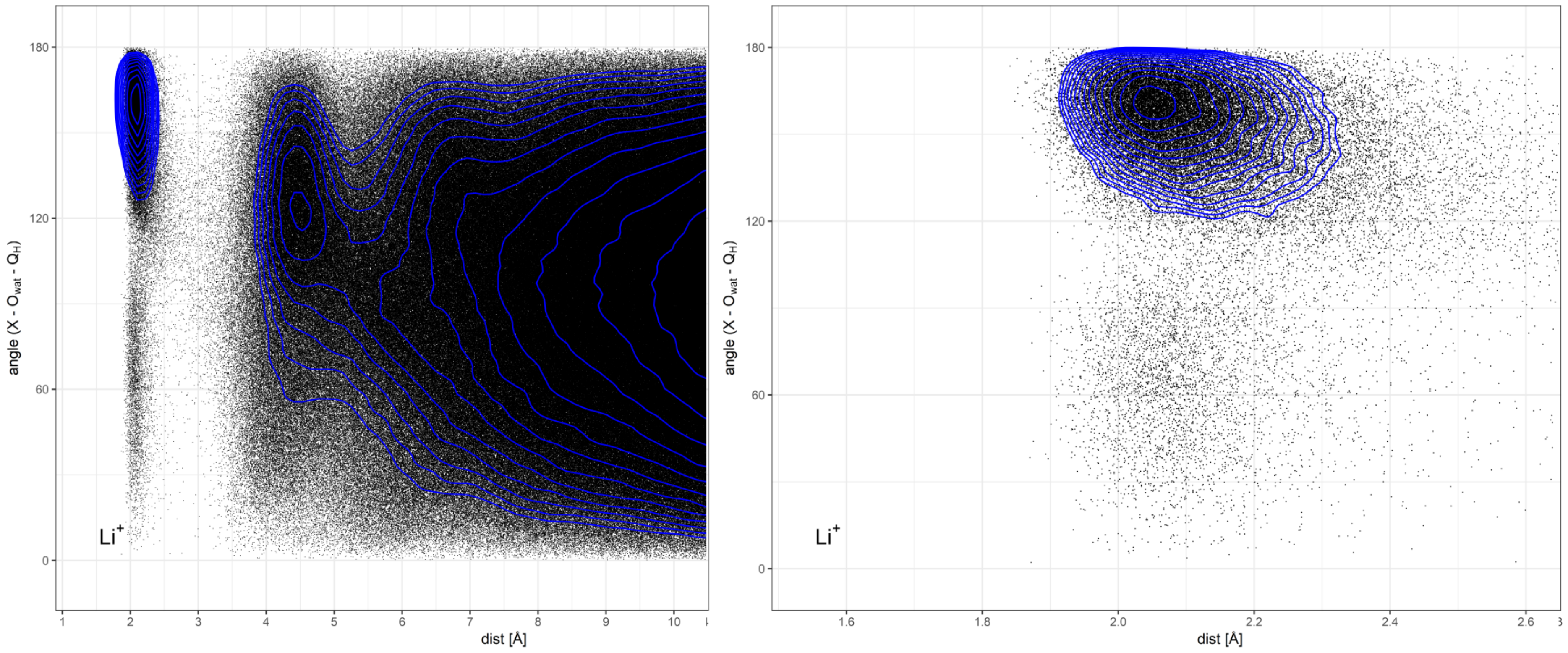

Supp. Figure 1B. Uncorrected density maps derived from 10^4 snapshots of 10ns MD simulations of the system, consisting of a Li^+ cation solvated by app 500 water molecules. Each dot represents the relative orientation of a single water molecule, defined by the distance between the center of mass of the water molecule (Q) to the solvated ion (dist) and the ion-O_{wat}-Q angle. The blue levels display the uncorrected water density in that space. The right panel zooms on the region corresponding to the first solvation shell.

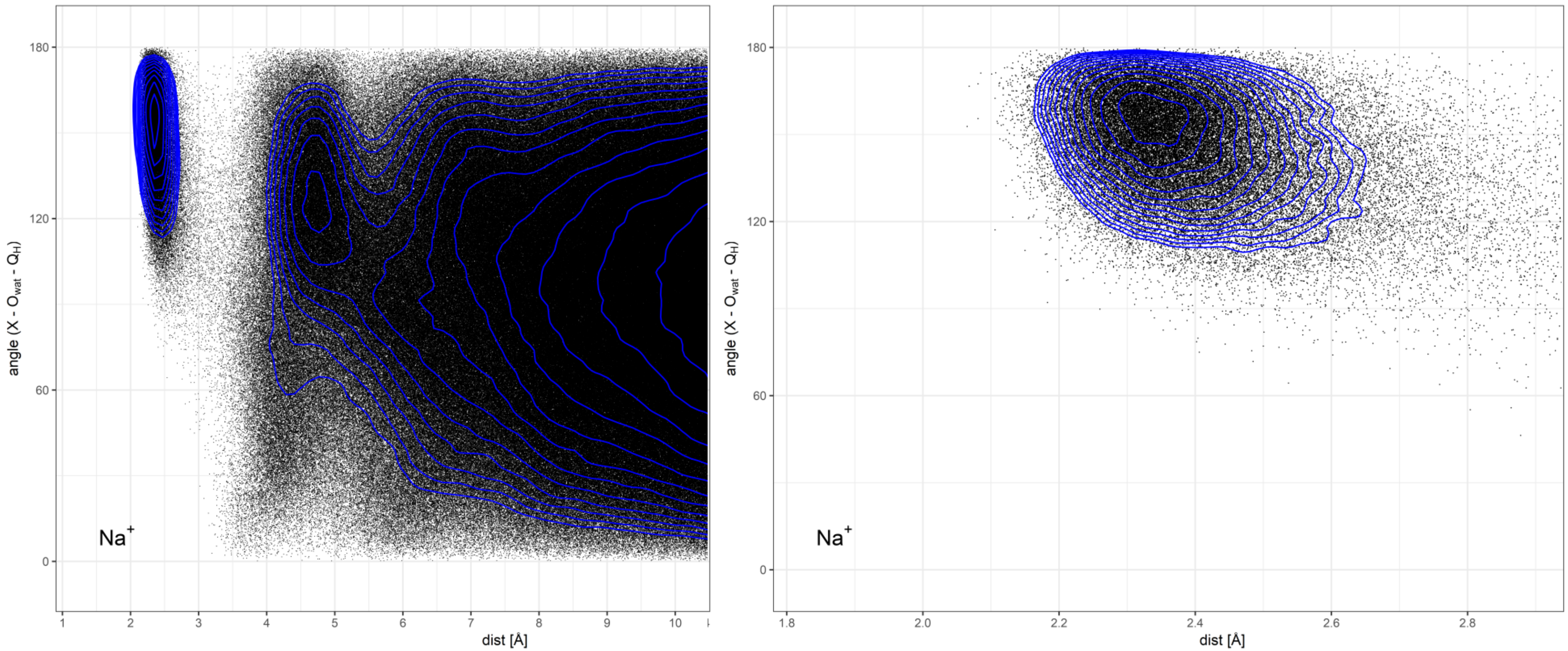

Supp. Figure 1C. Uncorrected density maps derived from 10^4 snapshots of 10ns MD simulations of the system, consisting of a Na^+ cation solvated by app 500 water molecules. Each dot represents the relative orientation of a single water molecule, defined by the distance between the center of mass of the water molecule (Q) to the solvated ion (dist) and the ion-O_{wat}-Q angle. The blue levels display the uncorrected water density in that space. The right panel zooms on the region corresponding to the first solvation shell.

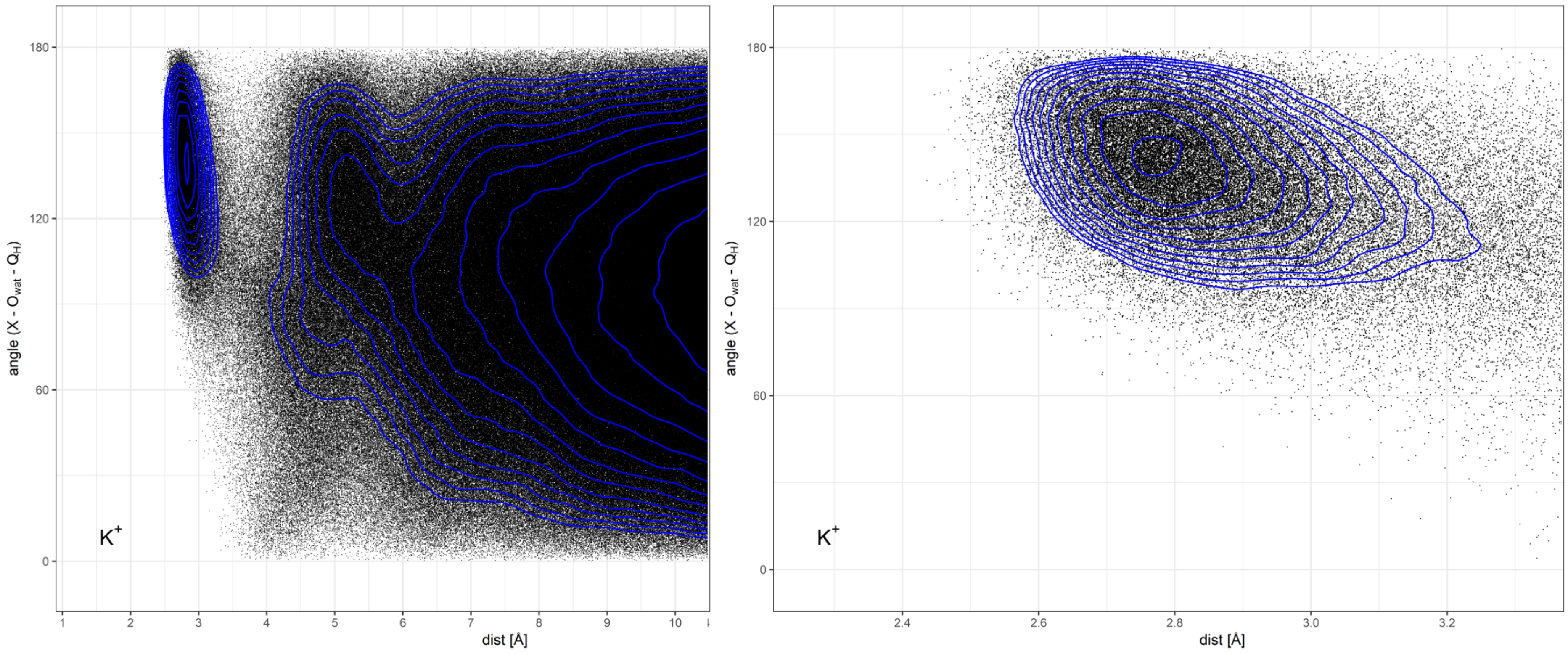

Supp. Figure 1D. Uncorrected density maps derived from 10^4 snapshots of 10ns MD simulations of the system, consisting of a K^+ cation solvated by app 500 water molecules. Each dot represents the relative orientation of a single water molecule, defined by the distance between the center of mass of the water molecule (Q) to the solvated ion (dist) and the ion-O_{wat}-Q angle. The blue levels display the uncorrected water density in that space. The right panel zooms on the region corresponding to the first solvation shell.

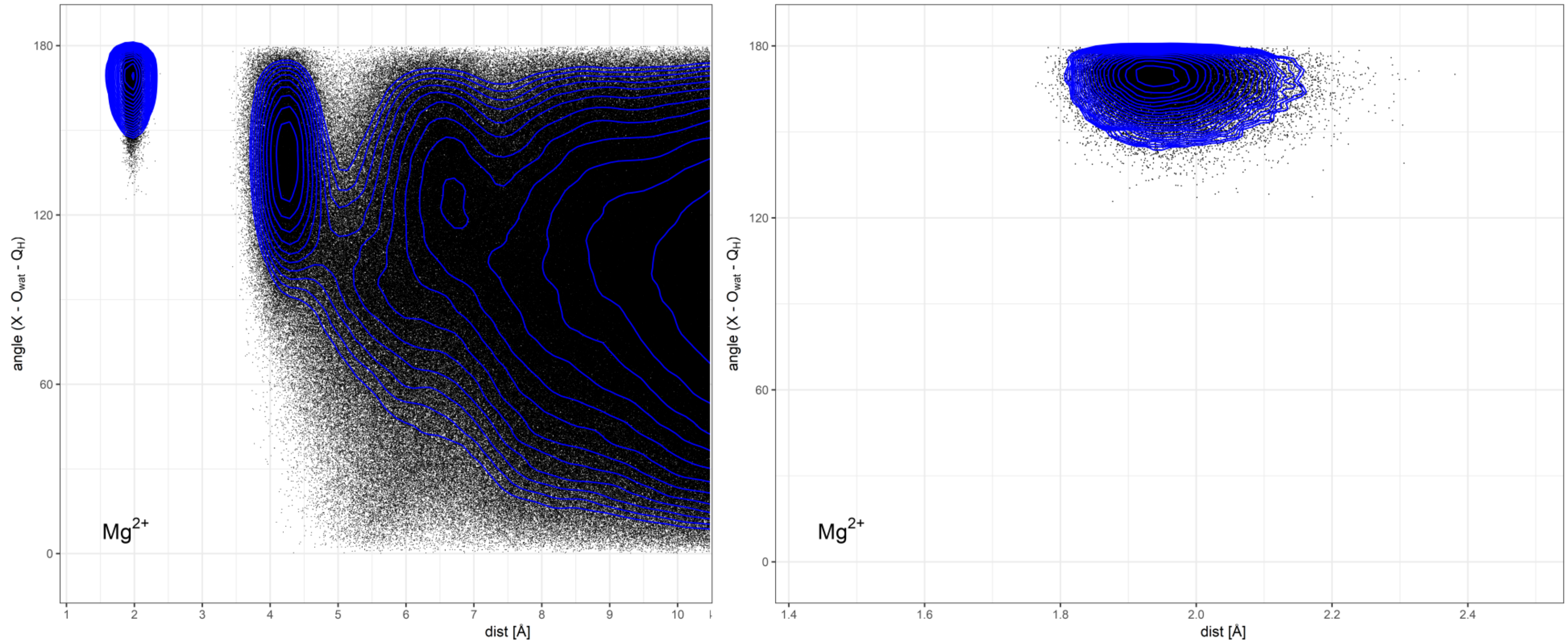

Supp. Figure 1E. Uncorrected density maps derived from 10^4 snapshots of 10ns MD simulations of the system, consisting of a Mg^{2+} cation solvated by app 500 water molecules. Each dot represents the relative orientation of a single water molecule, defined by the distance between the center of mass of the water molecule (Q) to the solvated ion (dist) and the ion-O_{wat}-Q angle. The blue levels display the uncorrected water density in that space. The right panel zooms on the region corresponding to the first solvation shell.

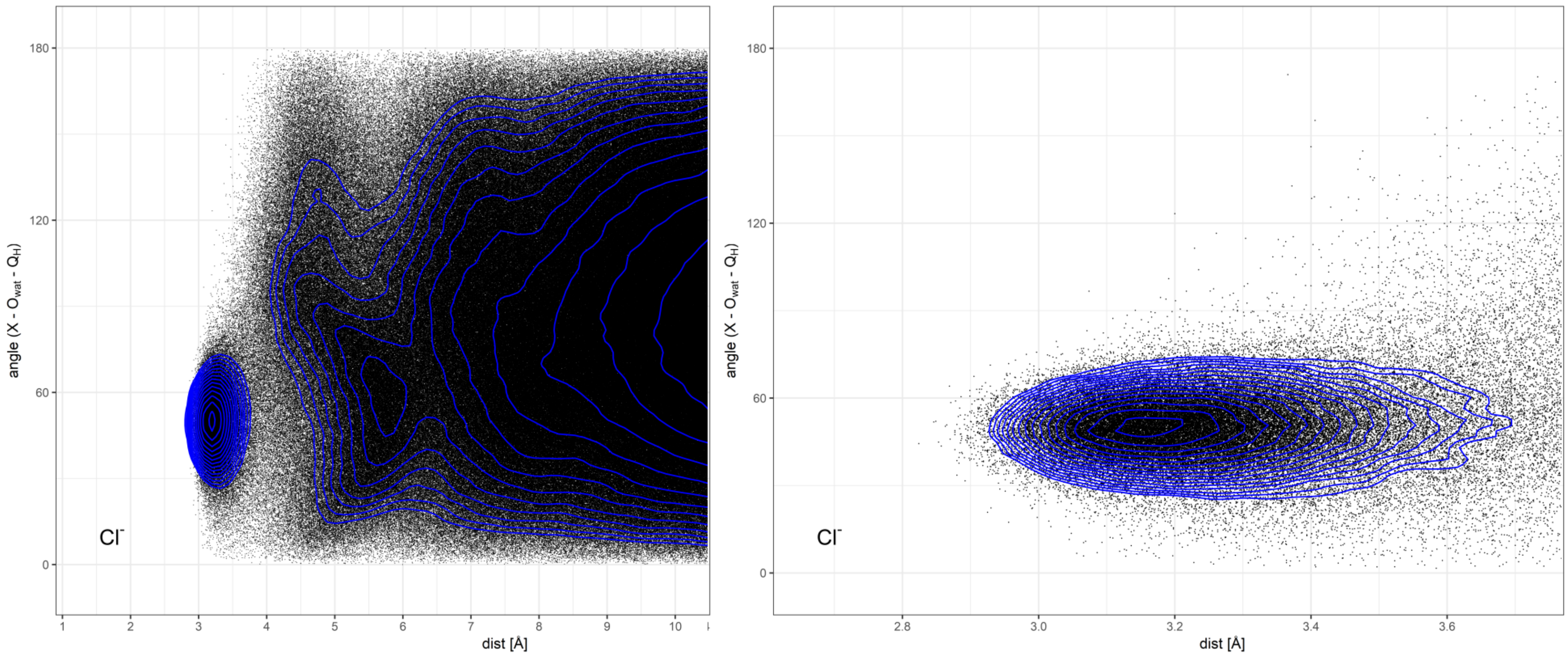

Supp. Figure 1F. Uncorrected density maps derived from 10^4 snapshots of 10ns MD simulations of the system, consisting of a Cl^- anion solvated by app 500 water molecules. Each dot represents the relative orientation of a single water molecule, defined by the distance between the center of mass of the water molecule (Q) to the solvated ion (dist) and the ion-O_{wat}-Q angle. The blue levels display the uncorrected water density in that space. The right panel zooms on the region corresponding to the first solvation shell.

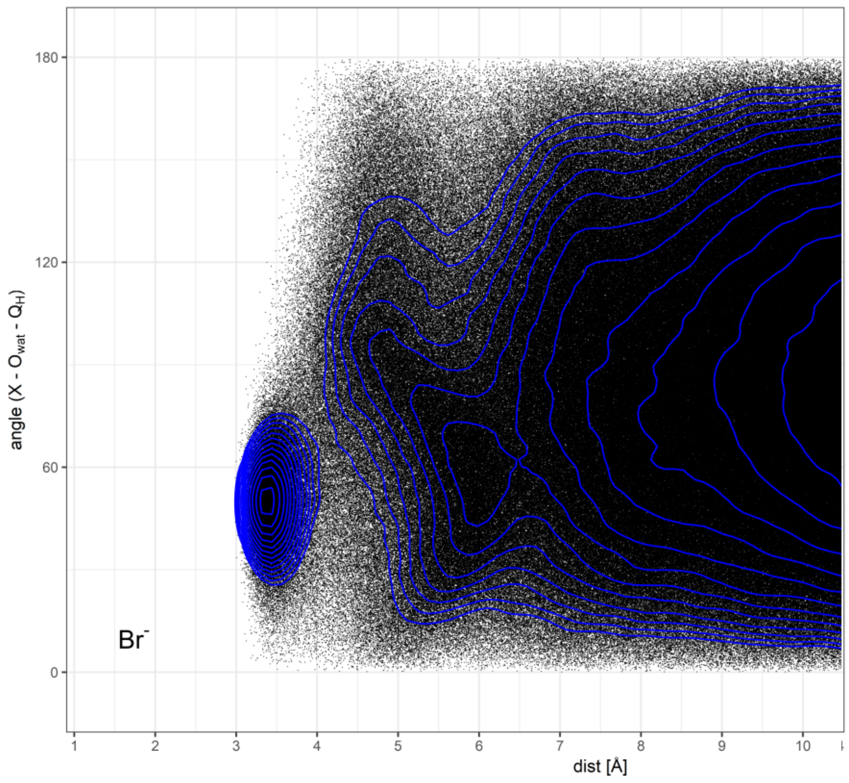

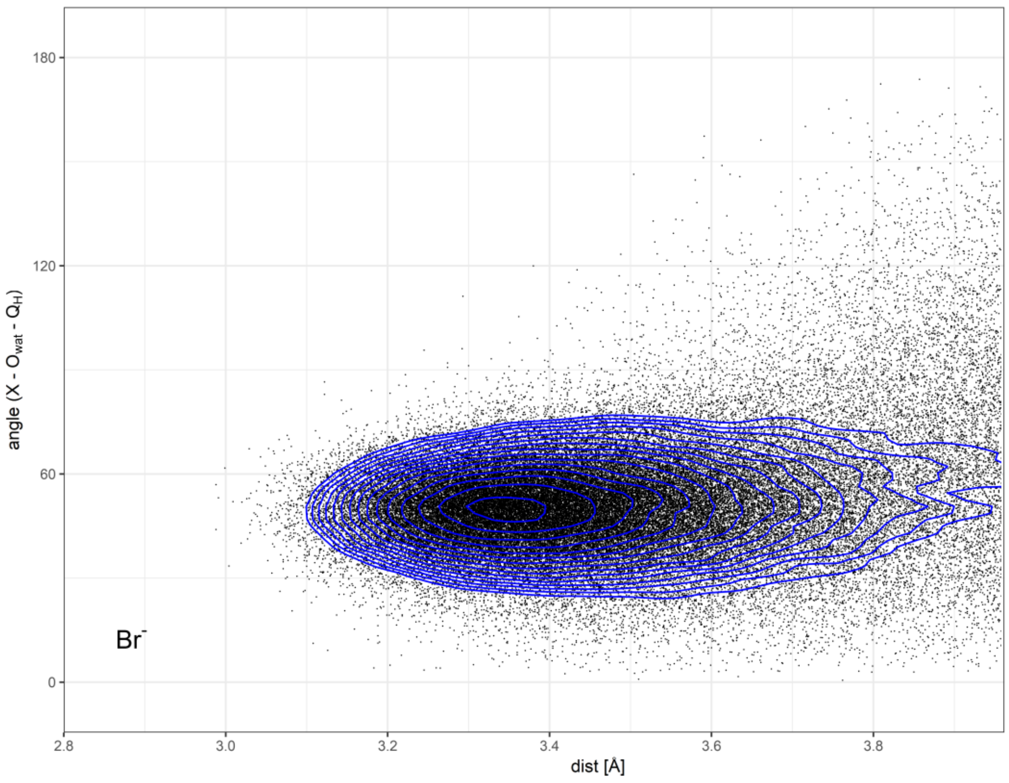

Supp. Figure 1G. Uncorrected density maps derived from 10^4 snapshots of 10ns MD simulations of the system, consisting of a Br^- cation solvated by app 500 water molecules. Each dot represents the relative orientation of a single water molecule, defined by the distance between the center of mass of the water molecule (Q) to the solvated ion (dist) and the ion-O_{wat}-Q angle. The blue levels display the uncorrected water density in that space. The right panel zooms on the region corresponding to the first solvation shell.

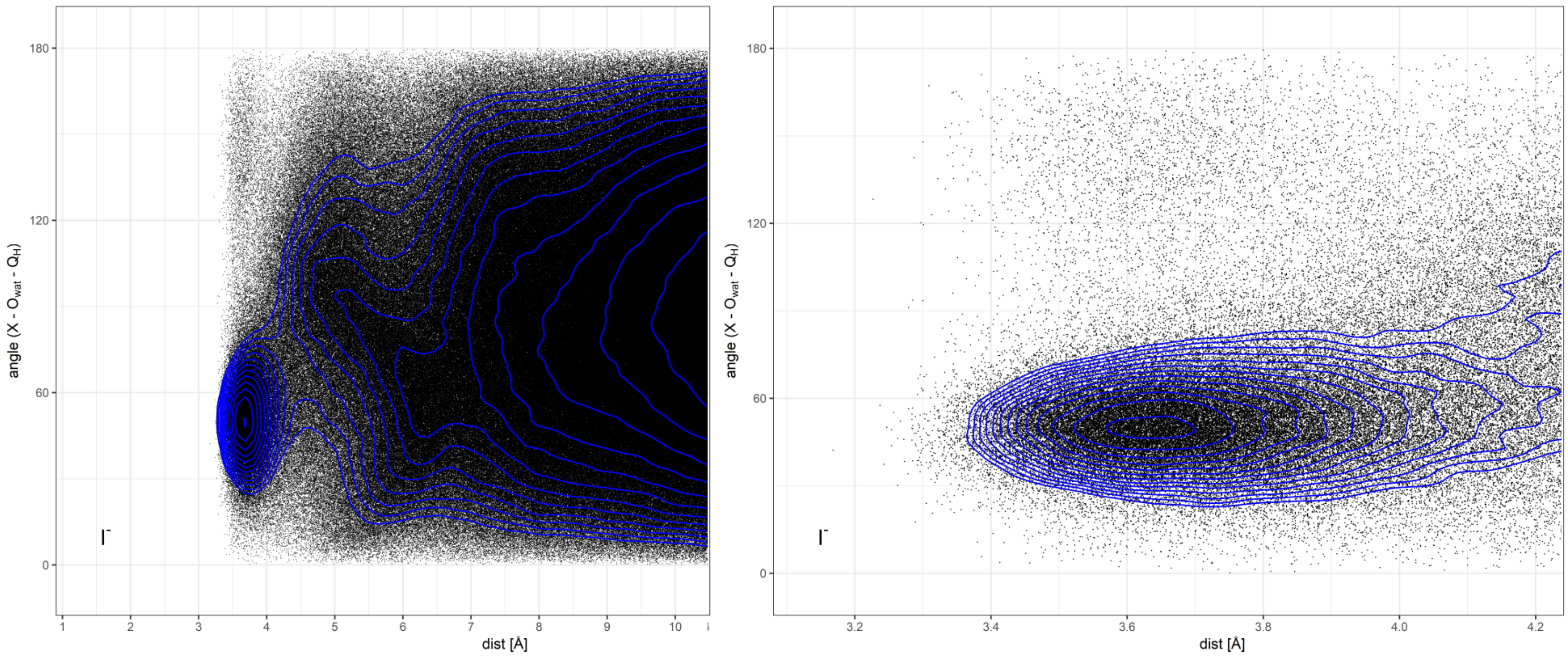

Supp. Figure 1H. Uncorrected density maps derived from 10^4 snapshots of 10ns MD simulations of the system, consisting of a I^- anion solvated by app 500 water molecules. Each dot represents the relative orientation of a single water molecule, defined by the distance between the center of mass of the water molecule (Q) to the solvated ion (dist) and the ion-O_{wat}-Q angle. The blue levels display the uncorrected water density in that space. The right panel zooms on the region corresponding to the first solvation shell.

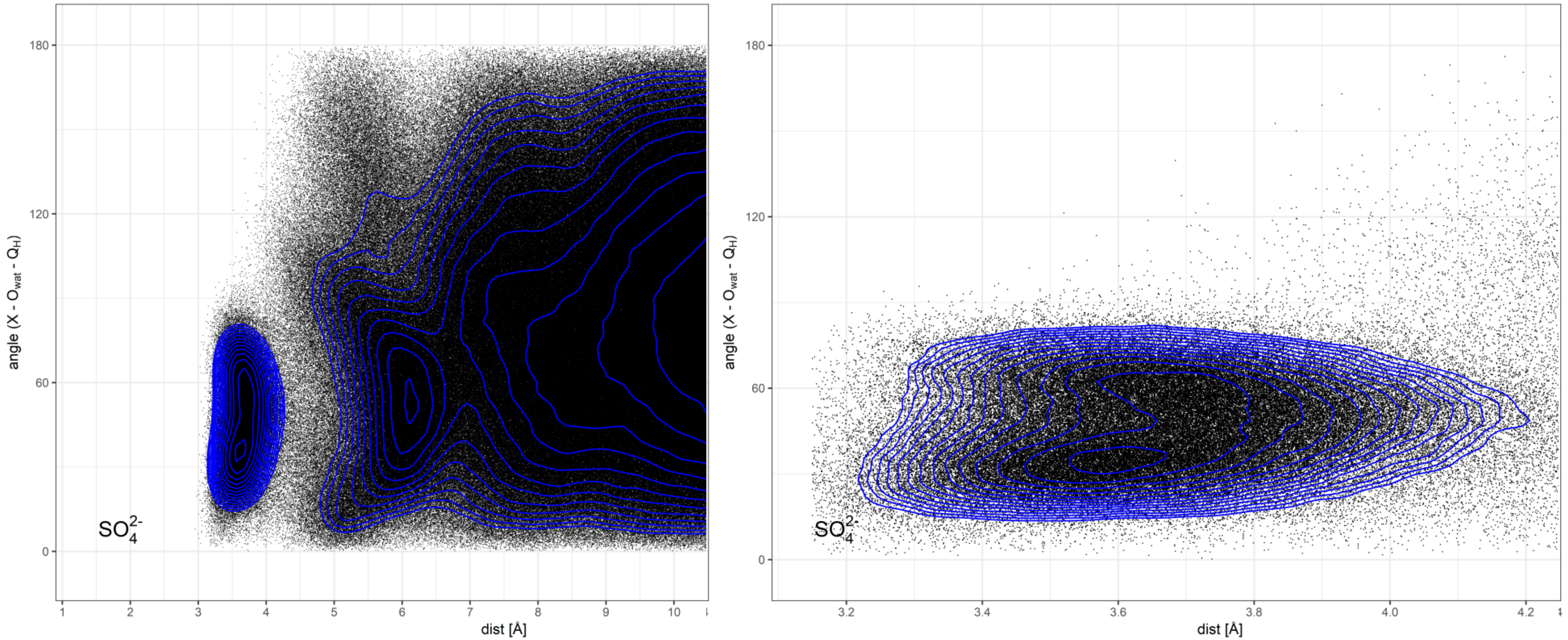

Supp. Figure 1I. Uncorrected density maps derived from 10^4 snapshots of 10ns MD simulations of the system, consisting of a SO_4^{2-} anion solvated by app 500 water molecules. Each dot represents the relative orientation of a single water molecule, defined by the distance between the center of mass of the water molecule (Q) to the solvated ion (dist) and the ion-O_{wat}-Q angle. The blue levels display the uncorrected water density in that space. The right panel zooms on the region corresponding to the first solvation shell.

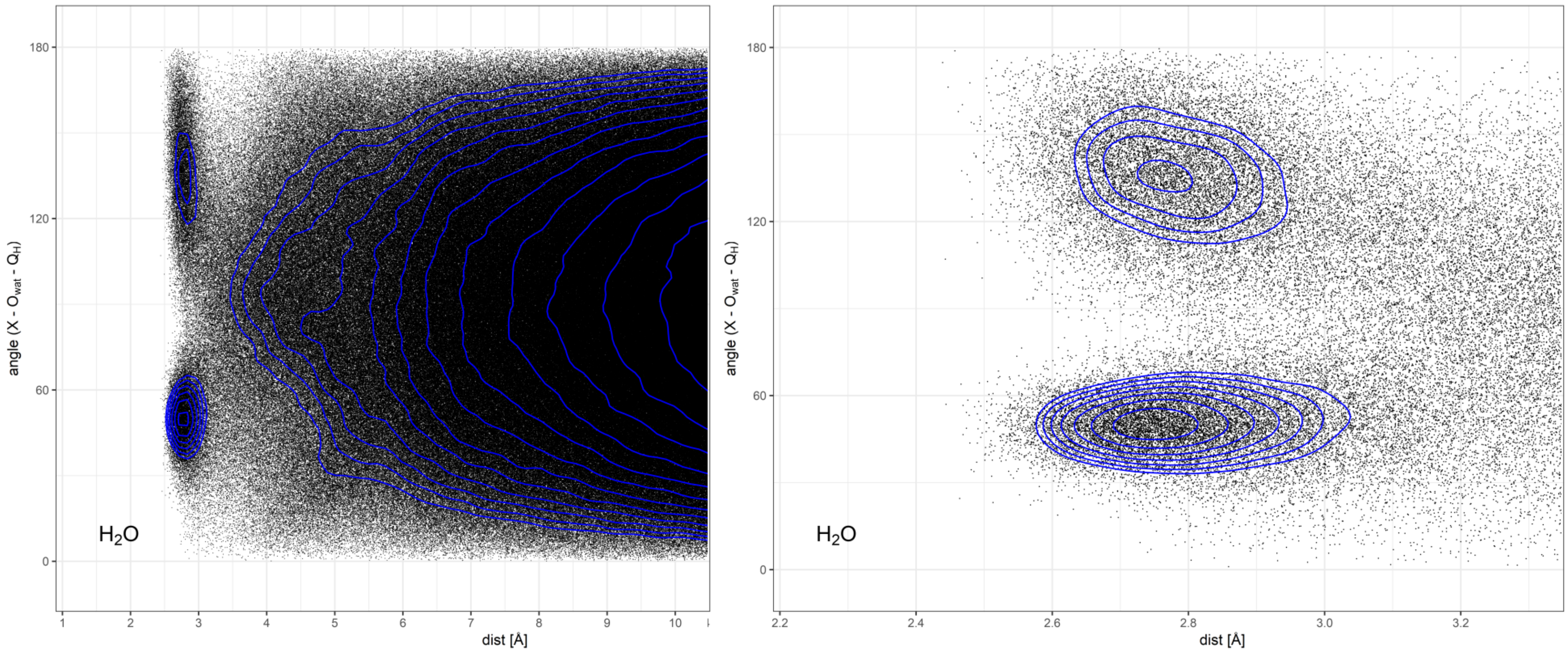

Supp. Figure 1J. Uncorrected density maps derived from 10^4 snapshots of 10ns MD simulations of the system, consisting of app 500 water molecules. Each dot represents the relative orientation of a single water molecule, defined by the distance between the center of mass of the water molecule (Q) to the oxygen atom of the central one (dist) and the O_{sel}-O_{wat}-Q angle. The blue levels display the uncorrected water density in that space. The right panel zooms on the region corresponding to the first solvation shell.
